# Estimation of the contribution of exports to the provincial economy: an analysis based on China’s multi-regional input–output tables

**DOI:** 10.1186/s40064-016-1803-7

**Published:** 2016-02-29

**Authors:** Sanmang Wu, Shantong Li, Yalin Lei

**Affiliations:** School of Humanities and Economic Management, China University of Geosciences, No. 29 Xueyuan Road, Haidian District, Beijing, 100083 People’s Republic of China; Key Laboratory of Carrying Capacity Assessment for Resource and Environment, Ministry of Land and Resource, Beijing, 100083 China; Development Research Center of State Council, Beijing, 100010 China

**Keywords:** Contribution of exports, Multi-regional input–output tables, Spillover effect

## Abstract

This paper developed an estimation model for the contribution of exports to a country’s regional economy based on the Chenery–Moses model and conducted an empirical analysis using China’s multi-regional input–output tables for 1997, 2002, and 2007. The results indicated that China’s national exports make significantly different contributions to the provincial economy in various regions, with the greatest contribution being observed in the eastern region and the smallest in the central region. The provinces are also subjected to significantly different export spillover effects. The boosting effect for the eastern provinces is primarily generated from local exports, whereas the western provinces primarily benefit from the export spillover effect from the eastern provinces. The eastern provinces, such as Guangdong, Zhejiang, Jiangsu, and Shanghai, are the primary sources of export spillover effects, and Guangdong is the largest source of export spillover effects for almost all of the provinces in China.

## Background

Foreign trade has played an important role in China’s rapid economic growth. This trade provides a broad and enormous market for China’s economy and accelerates China’s entry into the global division of labor and industrial system while simultaneously bringing capital, advanced technologies, and management experience to China’s economy, improving the efficiency of domestic resource utilization and configuration, and enhancing China’s international competitiveness. Especially after joining the World Trade Organization, China’s economy has gradually become more closely and profoundly integrated into the global economy. China’s total imports and exports reached $3.86676 trillion in 2012, which are 6.6 times China’s total imports and exports in 2001. China has surpassed Britain, France, Japan, and Germany to become the world’s second largest trading power after the United States.

Because of the increasingly important role of foreign trade, China’s economy is becoming increasingly closely connected with the global economy and is therefore more strongly affected by the global economy. The interaction between China’s economy and the global economy since the international financial crisis in 2008 is the best evidence for this connection. In the last two or three years, however, the sustained global economic downturn led to a strong economic decline in China.^1^ Therefore, an analysis of the contribution of exports to China’s economy has become an important area of research, and boosting China’s economy by promoting exports has become a concern for policymakers.

Unlike many other countries, China has vast territory with notably different geographical conditions and natural endowments across regions. As a result, each region’s participation in the national division of labor and position in the global industry chain is markedly different. Thus, changes in the global market have different impacts on China’s regional economy. The trade data show that nearly 90 % of China’s imports and exports occur in the ten provinces in the eastern region, but it is unclear whether this means that changes in the global market only affect the economies of the eastern provinces. This requires further analysis in the context of the constant integration of the domestic market. In short, properly understanding and estimating the contribution of exports to China’s regional economy is crucial for policymaking by both local and central governments.

The objective of the paper is to estimate the contribution of exports to the provincial economy in China. The used method of the paper is the measurement model for the contribution of exports to the regional economies of a country based on the Chenery–Moses model. This paper conducted an empirical analysis using the China’s multi-regional input–output tables for 1997, 2002, and 2007. The findings in the paper are as flowing:

First, national exports make significantly different contributions to provincial GDP in different regions in China.

Second, each province has a different source of contribution made by exports.

Third, Guangdong, Zhejiang, Jiangsu and Shanghai in the eastern region were the main source of export spillover effects for other provinces in China.

## Literature review

According to the existing literature, there are four primary types of quantitative research on the contribution of exports to the economy.

The first type uses direct foreign trade dependence [i.e., using exports as a percentage of Gross Domestic Product (GDP) to reflect the contribution of exports to the economy].^2^ Although this method is intuitive, it does not consider inherent economic linkages and cannot measure the contribution of exports to GDP.

The second type of quantitative research uses the national income identity to decompose GDP into consumption, investment and net exports using an elasticity formula to measure the contribution of net exports to economic growth. Typical examples of this type of research include studies by Chen et al. (2004). This method cannot be used to measure the contribution of exports. After comparing GDP data with net exports in previous years, Zhang and Hu (1999) determined that “net exports have ‘a negative correlation’ with GDP growth,” which also indicates that this method underestimates or mistakenly reflects the contribution of foreign trade.

The third type uses the econometrics regression model to analyze the contribution of exports to economic growth. Ghirmay’s (2001) study used time-series data from 15 low-income developing countries and a vector error correction model to examine the relationships among exports, investment and economic growth. Islam (1998) used a VEM model to study the relationship between export expansion and economic growth in 15 Southeast Asian countries. This method requires using time series data over a long period, so it is more suitable for mature economies in which exports make a stable contribution to economic growth. However, for a county with rapid foreign trade growth and a changing structure, such as China, this method cannot accurately measure the contribution of exports.

The fourth type of quantitative research uses input–output tables to measure the contribution of exports to the economy. The input–output model is a tool for analyzing the interdependence and mutual economic and technical constraints of various sectors of the national economy in the production process. This method uses the input–output identity and input–output multiplier model to measure the direct and indirect contributions of exports to the economy. The contribution takes place after the exports have a cyclic cumulative effect on the economy through the relationships among various production sectors of the national economy. Shen and Wu (2004) developed a method that uses input–output tables to measure the contribution of exports to the formation and growth of GDP. This method calculated and analyzed the contribution of each sector’s exports to China’s GDP growth and analyzed and revealed the reasons for the declining contribution rate of unit exports from 1997 to 2001. Many similar studies have been conducted, including those by Mattoo et al. (2013), Shen (2011) and Koopman et al. (Koopman et al. 2014). Compared with the first three quantitative analysis methods, this fourth method has the advantage of using a multiplier principle to satisfy the relationships among the national economic accounts and takes full account of the impact of economic structures. The input–output method thus measures both the macroeconomic effects and sector impacts. The disadvantage of this method is that it carries higher data requirements and requires the use of input–output tables.

Based on a comprehensive comparison of these four methods, we determined that the fourth method is the most suitable for measuring the contribution of China’s exports to the economy, especially the export contribution in a given year. However, it has not been found that the use of Multi-Regional Input–Output (MRIO) model to study the contribution of exports to a country’s regional economy. Wu et al. (2015) used the Single-Regional Input–Output (SRIO) model to measure the contributions of the four components of gross domestic product in various regions in China. The four components are consumption, international exports, inter-provincial and investment. The Single-Regional Input–Output model reflects the interaction between foreign trade and the internal economy, but it does not reflect the economic relations among different regions of the country. That means it does not reflect the spillover and feedback between the foreign trades in the different regions. Therefore, although the Single-Regional Input–Output model can estimate the direct contribution of the export to local economy. It is not able to estimate the indirect contribution of the exports to regional economy. For example, the Single-Regional Input–Output model can estimate the direct contribution of the export of a region to a region economy, but it cannot estimate the indirect contribution of the export of A region to B region economy. In addition, although many scholars have carried out analysis using Multi-Regional Input–Output (MRIO) models, these studies only focus on the measurement of economic relationships between regions instead of the contribution of exports to a regional economy. For example, Pan and Li (2007) also used IRIO tables and found that the spillover effect of a coastal area’s economic development on the inland area was not significant. In fact, it was even less significant than the inland area’s spillover effect on the coastal area. Many similar studies have been conducted, including studies by Shan and Wilson (2001) and Liu et al. (2012).

The existing literature shows that to measure the contribution of national exports to a regional economy more accurately, data and a model of interregional trade linkages are required. The MRIO model contains the inter-industrial linkages both within a region and between regions, so it can be applied to analyzing the contribution of exports to regional economy. Therefore, this study used the fourth method to develop China’s MRIO model, which was used to measure the contributions of national and regional exports to the regional economy.

## Multi-regional input–output model and data

### Multi-regional input–output model

In light of the theories of the input–output model, the IRIO tables/models proposed by Isard (1951) are an ideal tool for analyzing interregional economic linkages. These tables contain not only the economic linkages within a region but also detailed regional economic linkages. Input–output tables can clearly indicate the origins of goods in each region. In reality, however, it is difficult to collect such detailed trade data, especially trade coefficients. Therefore, few studies use the IRIO model proposed by Isard. To overcome the difficulties associated with data collection, Chenery (1953) and Moses (1955) proposed the MRIO model, which is also called the Chenery–Moses model or column coefficient model. This model assumes that different goods (intermediate inputs, final consumption, and investment in different sectors) in each region come from the same source.^3^ Therefore, only the interregional trade data are required to obtain the production and consumption locations of the tradable goods (instead of the sectors using them), whether for investment or final consumption. This greatly reduces the quantity of data needed, so this study used the MRIO model proposed by Chenery and Moses.^4^

Suppose that a country has *n* regions and *m* production sectors, and each industry only produces one product. The total demand for the product of sector *i* in region *r* can be expressed by the following equation:

1 $$\begin{array}{ll} {x_{i}^{r} = (t_{i1}^{r,1} + t_{i2}^{r,1} + \cdots + t_{i,12}^{r,1} + t_{i,m}^{r,1} + f_{i}^{r,1} )} \hfill & {\text{Total demand of region 1 for product i from region r}} \hfill \\ {\quad + (t_{i1}^{r,2} + t_{i2}^{r,2} + \cdots + t_{i,12}^{r,2} + t_{i,m}^{r,2} + f_{i}^{r,2} )} \hfill & {\text{Total demand of region 2 for product i from region r}} \hfill \\ {\quad + \cdots } \hfill & {} \hfill \\ {\quad + (t_{i1}^{r,r} + t_{i2}^{r,r} + \cdots + t_{i,12}^{r,r} + t_{i,m}^{r,r} + f_{i}^{r,r} )} \hfill & {\text{Total demand of region r for its own product i}} \hfill \\ {\quad + \cdots } \hfill & {} \hfill \\ {\quad + (t_{i1}^{r,n} + t_{i2}^{r,n} + \cdots + t_{i,12}^{r,n} + t_{i,m}^{r,n} + f_{i}^{r,30} )} \hfill & {\text{Total demand of region n for product i from region r}} \hfill \\ { \quad+ e_{i}^{r} } \hfill & {\text{Export demand of product i from region r}} \hfill \\ \end{array}$$

where *i* and *j* are the production sectors (*i, j* = 1,…, m), and *r* and *s* denote region (*r, s* = 1,…, n). $x_{i}^{r}$ is the total demand/output of products by sector *i* in region *r*^5^; $t_{i,j}^{r,s}$ is the intermediate input demand of sector *j* in region *s* for the product of sector *i* in region *r*; $f_{i}^{r,s}$ is the domestic final demand (including final consumption and investment) of region *s* for the products from sector *i* in region *r*; and $e_{i}^{r}$ is the export demand for the products of sector *i* in region *r*.

Equation 1 shows that a region’s product demand not only includes the intermediate input demand and final demand within the region but also the intermediate input demand and final demand of other domestic regions for the products. There is also the region’s export demand.

The most critical part of the regional input–output model is the O–D matrix of commodity flows (as shown in Table 1). The trade coefficients can be obtained through the O–D matrix (i.e., the composition of source areas of each product in each region and the composition of the destinations). Therefore, the MRIO model is used with the assumption that the products in the destination areas have the same source. The corresponding trade coefficients can be obtained by dividing the elements of the O–D matrix by the total number of the rows. Therefore, to determine the total demand of region *r* for product *i*, the proportion of this product provided by region *r* can be calculated ($c_{i}^{r,r}$), along with the proportion of the product provided by other regions ($c_{i}^{s,r}$).

**Table 1:** Flow matrix of product *i* (O–D matrix)

		Destination		
		1	…	n
Source	1	$z_{i}^{1,1}$	$z_{i}^{1,s}$	$z_{i}^{1,1}$
	…	…	…	…
	n	$z_{i}^{n,1}$	$z_{i}^{n,s}$	$z_{i}^{1,n}$
	Total	$d_{i}^{1}$	$d_{i}^{s}$	$d_{i}^{n}$
	$c_{i}^{r,r} = \frac{{z_{i}^{r,r} }}{{d_{i}^{r} }}$, $c_{i}^{s,r} = \frac{{z_{i}^{s,r} }}{{d_{i}^{r} }}$			

Through the regional input–output tables, the intermediate input technical coefficient of each region for the domestic products can be obtained ($a_{ij}^{r}$). This coefficient reflects the input demand of region *r* for various domestic products in producing every unit of product *j*, including the products from this region and from other domestic regions (Moses 1955). This effect can be expressed by the following equation:

$$a_{ij}^{r} = \frac{{t_{ij}^{ \bullet ,r} }}{{x_{j}^{r} }}$$

In the equation, the symbol $\bullet$ represents a summary of all the source areas. The trade coefficient and intermediate input coefficient are substituted into Eq. 1 to obtain the following equation:

2 $$\begin{aligned} x_{i}^{r} & = (c_{i}^{r,1} a_{i1}^{1} x_{i}^{1} + \cdots + c_{i}^{r,1} a_{i,m}^{1} x_{i}^{1} + c_{i}^{r,1} f_{i}^{ \bullet 1} ) \\ & \quad + \cdots \\ & \quad + (c_{i}^{r,n} a_{i1}^{n} x_{i}^{n} + \cdots + c_{i}^{r,n} a_{i,13}^{n} x_{i}^{n} + c_{i}^{r,n} f_{i}^{ \bullet n} ) \\ & \quad + e_{i}^{r} \\ \end{aligned}$$

where *i* = 1,…,13. Equation 2 can be rewritten in the form of the matrix as follows:

3 $$X = CAX + CF + E$$

In the matrix, X is the output matrix; C is the trade coefficient matrix; A is the matrix of the domestic intermediate input coefficient; F is the final demand matrix; and E is the export matrix. The specific elements of each matrix are as follows:

$${\text{X}} = \left[ {\begin{array}{*{20}c} {{\text{x}}^{1} } \\ {\begin{array}{*{20}c} {x^{2} } \\ \vdots \\ {{\text{x}}^{n} } \\ \end{array} } \\ \end{array} } \right],\,\,{\text{where}}\,\,\,\,{\text{x}}^{r} = \left[ {\begin{array}{*{20}c} {x_{1}^{r} } \\ {\begin{array}{*{20}c} {x_{2}^{r} } \\ \vdots \\ {x_{m}^{r} } \\ \end{array} } \\ \end{array} } \right],$$

and $x_{i}^{r}$ is the total output of sector *i* in region *r*.

$$C = \left[ {\begin{array}{*{20}c} {c^{1,1} } & \cdots & {c_{{}}^{1,n} } \\ \cdots & \cdots & \cdots \\ {c^{n,1} } & \cdots & {c^{n,n} } \\ \end{array} } \right],\,\,{\text{where}}\,\,c^{r,s} = \left[ {\begin{array}{*{20}c} {c_{1}^{r,s} } & 0 & 0 & 0 \\ 0 & {c_{2}^{r,s} } & 0 & 0 \\ 0 & 0 & \ddots & 0 \\ 0 & 0 & 0 & {c_{m}^{r,s} } \\ \end{array} } \right],$$

and $c_{i}^{r,s}$ is the trade coefficient (i.e., the proportion of the products of sector *i* in region *s* flowing from region *r* to the sector’s products flowing from all regions to region *s*).

$$A = \left[ {\begin{array}{*{20}c} {a^{1} } & 0 & 0 & 0 \\ 0 & {a^{2} } & 0 & 0 \\ 0 & 0 & \ddots & 0 \\ 0 & 0 & 0 & {a^{n} } \\ \end{array} } \right],\,\,\,{\text{where}}\,\,\,a^{r} = \left[ {\begin{array}{*{20}c} {a_{1,1}^{r} } & \cdots & {a_{1,m}^{r} } \\ \cdots & \cdots & \cdots \\ {a_{m,1}^{r} } & \cdots & {a_{m,m}^{r} } \\ \end{array} } \right],$$

and $a_{i,j}^{r}$ is the technical coefficient of the domestic intermediate input of sector *j* in region *r*.

$${\text{F}} = \left[ {\begin{array}{*{20}c} {{\text{f}}^{1} } \\ {\begin{array}{*{20}c} {{\text{f}}^{2} } \\ \vdots \\ {{\text{f}}^{\text{n}} } \\ \end{array} } \\ \end{array} } \right],\,\,{\text{where}}\,\,\, {\text{f}}^{r} = \left[ {\begin{array}{*{20}c} {f_{1}^{r} } \\ {\begin{array}{*{20}c} {f_{2}^{r} } \\ \vdots \\ {f_{\text{m}}^{r} } \\ \end{array} } \\ \end{array} } \right],$$

and $f_{i}^{r}$ is the final consumption demand (including consumption demand and investment demand) for the products of sector *i* in region *r*.

$${\text{E}} = \left[ {\begin{array}{*{20}c} {{\text{e}}^{1} } \\ {\begin{array}{*{20}c} {{\text{e}}^{2} } \\ \vdots \\ {{\text{e}}^{\text{n}} } \\ \end{array} } \\ \end{array} } \right],\,\,\,{\text{where}}\,\,\,\,{\text{e}}^{r} = \left[ {\begin{array}{*{20}c} {e_{1}^{r} } \\ {\begin{array}{*{20}c} {e_{2}^{r} } \\ \vdots \\ {e_{\text{m}}^{r} } \\ \end{array} } \\ \end{array} } \right],$$

and $e_{i}^{r}$ is the export demand for the products of sector *i* in region *r*.

Equation 3 can be further rewritten into Eq. 4:

4 $$X = CAX + CF + E \Rightarrow \left( {I - CA} \right)X = CF + E \Rightarrow X = \left( {I - CA} \right)^{ - 1} (CF + E)$$

Equation 4 can be used for simulation analysis, namely, the measurement of the contributions of various final demands (including domestic consumption, investment, and export) to total output. If the change in unit volume is used, the multiplier effect of various final demands can be calculated. The focus of this study is to measure the contributions of exports to a regional economy, so the export is separated from Eq. 4:

5 $$XE = \left( {I - CA} \right)^{ - 1} E$$

where XE is the total output contributed by the country’s regional exports. The value added rate of each sector is then introduced:

6 $$VAE = V\left( {I - CA} \right)^{ - 1} E$$

where VAE is the contribution of the country’s regional exports to the national value added value added, including direct and indirect contributions. The specific matrix elements are as follows:

$$V = \left[ {\begin{array}{*{20}c} {v^{1} } & 0 & 0 & 0 \\ 0 & {v^{2} } & 0 & 0 \\ 0 & 0 & \ddots & 0 \\ 0 & 0 & 0 & {v^{n} } \\ \end{array} } \right],\,\,{\text{where}}\,\,\,v^{r} = \left[ {\begin{array}{*{20}c} {v_{1}^{r} } & 0 & 0 & 0 \\ 0 & {v_{2}^{r} } & 0 & 0 \\ 0 & 0 & \ddots & 0 \\ 0 & 0 & 0 & {v_{m}^{r} } \\ \end{array} } \right],$$

and ${\text{v}}_{\text{i}}^{\text{r}}$ is the value added rate of sector *i* in region *r*.

$$VAE = \left[ {\begin{array}{*{20}c} {\begin{array}{*{20}c} {vae^{1} } \\ {vae^{2} } \\ \vdots \\ \end{array} } \\ {vae^{n} } \\ \end{array} } \right],\,\,\,{\text{where}}\,\,\,\,vae^{r} = \left[ {\begin{array}{*{20}c} {\begin{array}{*{20}c} {vae_{1}^{r} } \\ {vae_{2}^{r} } \\ \vdots \\ \end{array} } \\ {vae_{m}^{r} } \\ \end{array} } \right],$$

and $\hbox{vae}_{\text{i}}^{\text{r}}$ is the value added of sector *i* in region *r* contributed by the country’s regional exports.

The total national value added contributed by regional exports is

7 $${\text{VAE}} = \mathop \sum \limits_{r = 1}^{\text{n}} {\text{vae}}^{r}$$

The value added of region *r* contributed by national exports is

8 $$vae^{r} = \mathop \sum \limits_{i = 1}^{\text{m}} vae_{i}^{r}$$

However,

9 $${\text{VAE }}=\mathop \sum \limits_{r = 1}^{\text{n}} \mathop \sum \limits_{i = 1}^{\text{m}} vae_{i = 1}^{r}$$

That is, the value added contributed by national exports can be decomposed into the sum of the value added of different regions contributed by national exports. In addition, through further decomposition, the contribution of the exports of region *r* to the national value added and to the value added of region *s* can be obtained.

10 $$VAE^{ \bullet ,r} = V\left( {I - CA} \right)^{ - 1} E^{r}$$

$VAE^{ \bullet ,r}$ is the contribution of region *r*’s exports to the regional value added of the country. $E^{r}$ is the export matrix of region *r*. The forms of the matrix elements of $VAE^{ \bullet ,r}$ are as follows:

11 $$VAE^{ \bullet ,r} = \left[ {\begin{array}{*{20}c} {{\text{vae}}^{1,r} } \\ {\begin{array}{*{20}c} {{\text{vae}}^{2,r} } \\ \vdots \\ {{\text{vae}}^{{{\text{n}},r}} } \\ \end{array} } \\ \end{array} } \right]$$

$VAE^{{{\text{s}}, {\text{r}}}}$ is the value added of region *s* contributed by the exports of region *r*. The forms of the matrix elements of ${\text{vae}}^{{{\text{s}}, {\text{r}}}}$ are as follows:

12 $${\text{vae}}^{{{\text{s}}, {\text{r}}}} = \left[ {\begin{array}{*{20}c} {vae_{1}^{s,r} } \\ {\begin{array}{*{20}c} {vae_{2}^{s,r} } \\ \vdots \\ {vae_{\text{m}}^{s,r} } \\ \end{array} } \\ \end{array} } \right]$$

$vae_{i}^{e,r}$ is the value added of sector *i* in region *s* contributed by the exports of region *r*. Therefore, the total value added of region *s* contributed by the exports of region *r* is

13 $${\text{vae}}^{{{\text{s}}, {\text{r}}}} = \mathop \sum \limits_{i}^{\text{m}} vae_{i}^{s,r}$$

Equation 13 can be used to calculate the direct and indirect contributions of exports to the value added of a particular region. When r = s, ${\text{vae}}^{{{\text{s}}, {\text{s}}}}$ is the direct contribution of the exports of region *s* to its value added; when r ≠ s, ${\text{vae}}^{{{\text{s}}, {\text{r}}}}$ is the indirect contribution of the exports of region *r* to the value added of region *s*.

### Data

The MRIO model was developed from the MRIO tables. The Development Research Center of the State Council of China cooperated with the National Bureau of Statistics of China and other collaborators several times to develop China’s MRIO tables for 1997, 2002, and 2007 (Xu and Li 2008; Li et al. 2010; Li and Xu 2012). The Institute of Geographic Sciences and Natural Resources Research of the Chinese Academy of Sciences developed regional input–output tables for 30 provinces in 2007 (Liu et al. 2012).

This paper used China’s MRIO tables for 1997, 2002, and 2007, which were jointly developed by the Development Research Center of the State Council of China and the National Bureau of Statistics of China (Xu and Li 2008; Li et al. 2010; Li and Xu 2012). The MRIO table for 2007 is the most recent MRIO table for China. These MRIO tables cover 30 provinces and 42 sectors for each region (no input–output tables are available for Tibet, so Tibet was not included). To facilitate the paper, the 42 sectors were combined into 13 sectors. For details, refer to Table 2.

**Table 2:** Sector classification of multi-regional input–output tables in this paper

A01	Agriculture, forestry, animal husbandry, and fishery
A02	Mining
A03	Food, textiles, clothing, wood, and paper-making
A04	Petrochemical
A05	Building materials
A06	Metal smelting and rolling and metal products
A07	Other manufacturing industries
A08	Electricity, gas, and water
A09	Building
A10	Transportation, postal service, and telecommunications
A11	Commerce, accommodation, and catering
A12	Finance and real estate
A13	Other services

## Calculation results and analysis

### Analysis of the contribution of national exports to provincial GDP

Table 3 shows the calculation of the contribution of national exports to provincial GDP in 2007 (including direct and indirect contributions) based on the MRIO tables. In Table 3, the second column shows the GDP of each province, and the third column and fourth column show the value added for each province contributed by national exports and the province’s percentage of GDP, respectively (i.e., the contribution of national exports to each province). The fifth column and sixth column exhibit each province’s total exports and their percentages of GDP (i.e., the export dependence of each province).

**Table 3:** Contribution of national exports to each province in 2007 (in 100 million yuan)

Region	GDP	Each province’s value added contributed by national exports (VAE)	Contribution of national exports to provincial GDP (VAE/GDP) (%)	Total exports of each province	Foreign export dependence of each province (%)
*Eastern provinces*					
Beijing	9579	2579	27	4363	46
Tianjin	5050	1564	31	2248	45
Hebei	13,778	2736	20	1388	10
Shanghai	12,189	5143	42	11,220	92
Jiangsu	26,508	9044	34	13,508	51
Zhejiang	18,839	6009	32	9590	51
Fujian	9249	2840	31	3829	41
Shandong	25,575	6592	26	6765	26
Guangdong	30,843	13,118	43	28,666	93
Hainan	1203	181	15	184	15
*Central provinces*					
Shanxi	5733	1020	18	569	10
Anhui	7335	1152	16	650	9
Jiangxi	5500	542	10	408	7
Henan	15,012	1806	12	679	5
Hubei	9402	889	9	599	6
Hunan	9200	859	9	448	5
*Western provinces*					
Inner Mongolia	6288	1119	18	268	4
Guangxi	5959	784	13	435	7
Chongqing	4179	405	10	304	7
Sichuan	10,505	751	7	497	5
Guizhou	2772	355	13	146	5
Yunnan	4758	555	12	182	4
Shaanxi	5575	987	18	386	7
Gansu	2753	521	19	442	16
Qinghai	797	72	9	68	9
Ningxia	899	146	16	122	14
Xinjiang	3596	653	18	278	8
*Northeastern provinces*					
Liaoning	11,194	2422	22	2466	22
Jilin	5407	684	13	274	5
Heilongjiang	7071	1142	16	383	5

Table 3 indicates that the national exports made significantly different contributions to each province’s GDP in China. China’s national exports made greater contributions to the GDP of most of the eastern provinces. The contribution was the greatest to Guangdong Province, whose value added contributed by national exports in 2007 reached 1311.8 billion yuan, accounting for 43 % of Guangdong’s GDP. National exports made smaller contributions to the GDP of the central provinces, such as Hunan and Hubei Provinces, whose value added contributed by national exports only accounted for approximately 9 % of their provincial GDPs. Although the western provinces are the farthest from the export ports, the contribution of national exports to these provinces’ GDP was not the smallest. The average contribution in the western provinces was greater than the contribution in the central provinces (the arithmetic mean of the percentages of the value added of the 11 western provinces contributed by national exports was 7.8 %, whereas that of the 6 central provinces was 7 %).

Table 3 also shows the foreign export dependence of each province. The data in Table 3 indicate that the foreign export dependence of most of the eastern provinces was relatively higher. The foreign export dependence of Guangdong Province was the highest, reaching 93 % in 2007. To some extent, this explains why national exports made such a great contribution to the GDP of the eastern provinces. By comparing the foreign export dependence of each eastern province with the contribution of national exports to their GDP, however, we observed that the contribution of national exports to the eastern provinces’ GDP was far less than their foreign export dependence. For example, the foreign export dependence of Guangdong Province was 93 %, whereas its value added contributed by national exports accounted for 43 % of its GDP. There are two important reasons for this: first, a large part of China’s foreign trade belongs to the processing trade (i.e., exports require the import of a large number of intermediate products), and the domestic value added rate is low. Therefore, although the foreign export dependence is higher, the percentage of the value added contributed by exports is relatively low. The economic relationships between the regions lead to high export spillover effects in the eastern provinces; in other words, the exports of these provinces require the purchase of intermediate raw materials from inland provinces, which boosts the GDPs of the inland provinces. This is why foreign export dependence cannot be used to measure the contribution of exports to provincial GDP. In fact, the contribution of exports to the eastern provinces’ economies tends to be overestimated when using foreign export dependence.

In other regions, the foreign export dependence of the central and western provinces is significantly lower than that of eastern provinces. The foreign export dependence of the central provinces was the lowest and accounted for only 6.39 %. Above, we noted that the contribution of exports to the western provinces’ economies was not the lowest, but was higher than the contribution of exports to the central provinces’ economies. Considering that the foreign export dependence of the western region was the lowest, it can be inferred that the economic relationships between the western and eastern provinces are closer than the economic relationships between these regions and the central regions. By comparing the western provinces’ foreign export dependence with their contribution to exports, we found that for the western provinces, the contribution of national exports to GDP was significantly higher than the foreign export dependence. For example, Yunnan Province’s foreign export dependence was only 4 % in 2007, whereas the contribution of national exports to its GDP reached 12 %. The contributions of national exports to the GDP of Inner Mongolia, Guangxi, Chongqing, Sichuan, Guizhou, Yunnan, Shaanxi, Gansu, Qinghai, Ningxia, and Xinjiang were substantially higher than their foreign export dependence. Therefore, for the western provinces, the contribution of exports to their economies will be underestimated when using foreign export dependence because the spillover effects of export production in other regions are not considered.

The changes in the contribution of national exports to provincial economies (Table 4) indicated that generally, the contribution of national exports to provincial economies gradually increased. The increasing trend was especially pronounced from 2002 to 2007. For example, Shanghai’s value added contributed by national exports as a percentage of GDP increased from 28 % in 1997 to 42 % in 2007. This suggests that exports have an increasing impact on China’s provincial economies, and China’s provinces are gradually becoming integrated into the global market, either directly or indirectly. In addition, the data in Table 3 indicate that the foreign export dependence of the eastern provinces was generally lower than the contribution of national exports to their GDP, whereas the foreign export dependence of the central and western provinces was generally higher than the contribution of national exports to their GDP.

**Table 4:** Changes in the contribution of national exports to provincial economies

Region	Foreign export dependence of each province (%)			Contribution of national exports to the GDP of each province (%) (VAE/GDP)		
	1997	2002	2007	1997	2002	2007
*Eastern provinces*						
Beijing	35	19	46	23	16	27
Tianjin	36	49	45	24	28	31
Hebei	6	6	10	12	11	20
Shanghai	41	56	92	28	33	42
Jiangsu	19	31	51	19	23	34
Zhejiang	20	31	51	19	23	32
Fujian	33	30	41	25	25	31
Shandong	16	16	26	16	16	26
Guangdong	91	68	93	43	35	43
Hainan	19	7	15	16	11	15
*Central provinces*						
Shanxi	12	8	10	16	11	18
Anhui	6	6	9	11	11	16
Jiangxi	6	3	7	8	6	10
Henan	4	3	5	7	6	12
Hubei	6	4	6	8	6	9
Hunan	5	4	5	8	6	9
*Western provinces*						
Inner Mongolia	6	4	4	11	8	18
Guangxi	9	6	7	12	9	13
Chongqing	4	5	7	9	8	10
Sichuan	5	5	5	7	6	7
Guizhou	6	4	5	8	8	13
Yunnan	8	4	4	10	6	12
Shaanxi	9	0	7	10	5	18
Gansu	4	10	16	7	9	19
Qinghai	7	9	9	9	8	9
Ningxia	9	3	14	11	6	16
Xinjiang	4	6	8	9	10	18
*Northwestern provinces*						
Liaoning	21	18	22	18	17	22
Jilin	10	7	5	12	12	13
Heilongjiang	12	5	5	15	10	16

### Analysis of total spillover effect of exports

**Fig. 1:**
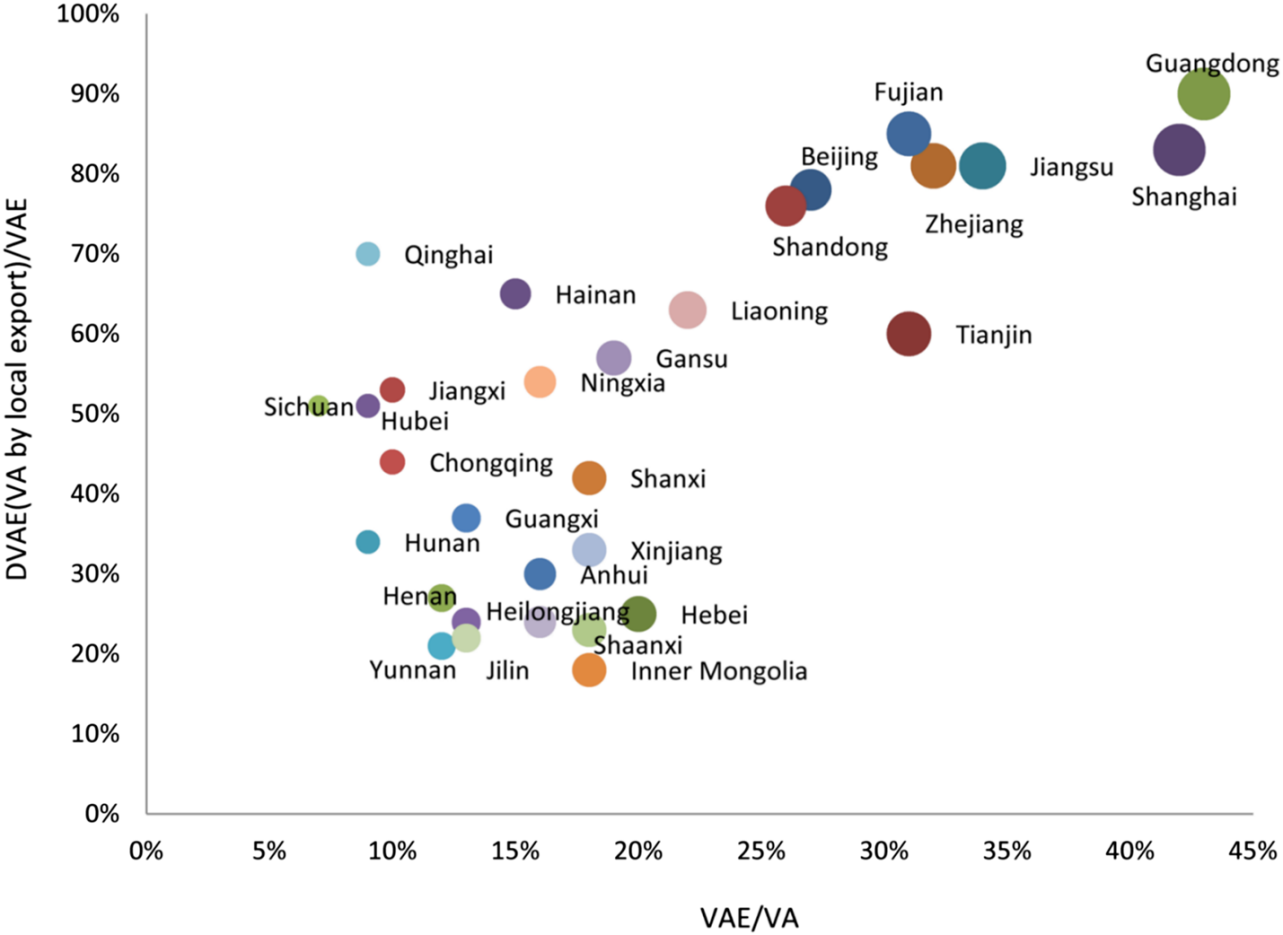
Direct contribution of exports to provincial economy in 2007. *Note:* The *vertical axis* represents the percentage of each province’s value added contributed by local exports compared to the percentage of the value added contributed by national exports; the *horizontal axis* represents the percentage of each province’s value added contributed by national exports to the GDP

In the previous section, we analyzed the contribution of national exports to each province’s GDP. Part of the contribution was made by local exports, also known as direct contributions; the other part of the contribution was made by the exports of other provinces, known as the spillover effects of interregional exports. Figure 1 shows the direct contribution of exports to each province’s economy in 2007 [i.e., the percentage of each province’s value added contributed by local exports to those contributed by national exports (the vertical axis)]. As shown in Fig. 1, the contribution of the exports of the eastern provinces was high and accounted for approximately 80–90 % of their total contributions. For example, the value added of Guangdong Province contributed by local exports accounted for 90 % of its value added contributed by national exports in 2007. The contribution of the exports of the central and western provinces was low, however, especially in certain western provinces that are rich in resources. For example, Shanxi, Inner Mongolia, and Shaanxi are China’s main coal-producing areas and produced a total of 9.94, 9.6, and 4.63 tons of coal in 2012, respectively, ranking first, second, and third in China in terms of coal production. The value added of Shanxi, Inner Mongolia, and Shaanxi contributed by local exports accounts for only 10–20 % of the value added contributed by national exports. This indicates that the contribution of exports to these provinces’ GDP stems primarily from the spillover effects of interregional exports.

To show the different sources of contributions to each province’s economy, the changes in the indirect contribution of exports to each province’s economy in 1997, 2002, and 2007 are listed in Table 5. Table 5 shows that each province’s value added contributed indirectly by exports increased. This indicates that the level of integration of China’s domestic market is improving and the economic connections between regions are strengthening. At the regional level, however, the indirect contribution of exports to the central, western, and northeastern provinces has been consistently high, whereas the indirect contribution of exports to the eastern provinces has been low. In addition, Table 4 shows that the indirect contribution of exports to the western provinces, such as Inner Mongolia, Shaanxi, Guizhou and Yunnan, increased year by year. For example, the indirect contribution of exports to Inner Mongolia increased from 60 % in 1997 to 63 % in 2002 and 82 % in 2007. This indicates that exports made an increasing contribution to the western provinces’ economies through the export spillover effect of other provinces [i.e., other provinces contribute to these provinces’ economic growth by purchasing raw materials (or energy) from them for production and export]. Therefore, the western provinces usually act as suppliers of raw materials to other provinces. Considering that the eastern provinces’ exports made a greater direct contribution, the pattern of regional economic development in China can be visualized. The eastern provinces are gradually integrated into the global industrial chain through the processing trade. Most of the western provinces do not integrate into the global industrial chain, but instead become the suppliers of raw materials for the eastern provinces. This suggests that the level of integration of China’s domestic market is improving and the economic connections between provinces are tightening; however, this leads to a greater regional development gap (Liu and Zhang 2008).

**Table 5:** Changes in the indirect contribution of exports to provincial economies

Region	Each province’s value added contributed by the exports of other provinces (100 million yuan)			Indirect contribution of exports to each province’s economy^a^ (%)		
	1997	2002	2007	1997	2002	2007
*Eastern provinces*						
Beijing	63	241	578	16	34	22
Tianjin	83	133	622	28	22	40
Hebei	324	442	2055	68	67	75
Shanghai	183	262	869	20	14	17
Jiangsu	468	333	1718	38	14	19
Zhejiang	186	423	1137	22	23	19
Fujian	114	96	416	15	9	15
Shandong	281	380	1607	26	24	24
Guangdong	159	265	1291	5	6	10
Hainan	21	36	63	27	55	35
*Central provinces*						
Shanxi	107	94	596	47	36	58
Anhui	192	292	809	65	72	70
Jiangxi	66	88	256	48	60	47
Henan	160	192	1316	56	56	73
Hubei	133	125	436	47	50	49
Hunan	119	145	564	52	56	66
*Western provinces*						
Inner Mongolia	69	100	919	60	63	82
Guangxi	115	126	491	48	57	63
Chongqing	81	116	225	68	70	56
Sichuan	101	112	365	43	40	49
Guizhou	34	62	269	53	64	76
Yunnan	68	83	436	41	56	79
Shaanxi	56	109	761	42	67	77
Gansu	36	40	223	64	35	43
Qinghai	10	9	22	56	33	30
Ningxia	12	16	68	51	68	46
Xinjiang	67	96	435	68	58	67
*Northeastern provinces*						
Liaoning	129	228	888	20	25	37
Jilin	68	186	532	40	67	78
Heilongjiang	176	224	863	44	59	76

^a^The ratio of the province’s total value added contributed by the exports of other provinces to the value added contributed by national exports

A province will not only be affected by the export spillover effects of other provinces but also produces a spillover effect on other provinces. Table 6 shows each province’s export spillover effects on other provinces in 1997, 2002, and 2007. Based on the value added of each province contributed by the export spillover effect, the eastern provinces’ export spillover effects was the greatest. In particular, Guangdong Province had the greatest export spillover effect, and the value added of other provinces contributed by their exports reached 607.8 billion yuan in 2007. The export spillover effect of the western provinces was relatively smaller, and the value added of other provinces contributed by the exports of western provinces was essentially less than 10 billion yuan in 2007. This is related to the larger amount of exports from the eastern provinces and the position of each province in the industrial chain. The eastern provinces are mostly located downstream in the industrial chain, so their exports make a greater contribution; the central and western provinces are upstream in the industrial chain, so their exports make a smaller contribution.

**Table 6:** Changes in each province’s export spillover effects

Region	Value added of export spillover (100 million yuan)^a^			Proportion of export spillover^b^ (%)		
	1997	2002	2007	1997	2002	2007
*Eastern provinces*						
Beijing	127	237	1211	27	34	38
Tianjin	136	306	858	39	40	48
Hebei	77	115	562	33	34	45
Shanghai	334	518	2234	31	24	34
Jiangsu	340	495	2563	31	19	26
Zhejiang	173	784	3148	20	36	39
Fujian	174	185	868	22	16	26
Shandong	172	269	1019	18	18	17
Guangdong	1457	1430	6078	33	24	34
Hainan	21	11	18	28	28	13
*Central provinces*						
Shanxi	45	25	76	27	13	15
Anhui	53	69	240	34	38	41
Jiangxi	21	21	90	22	27	24
Henan	32	34	145	20	18	23
Hubei	36	27	103	19	18	19
Hunan	38	26	117	25	19	28
*Western provinces*						
Inner Mongolia	18	14	49	28	20	20
Guangxi	42	46	102	25	33	26
Chongqing	18	35	96	32	42	35
Sichuan	27	35	79	16	17	17
Guizhou	15	10	47	34	22	36
Yunnan	30	17	35	24	20	23
Shaanxi	37	2	128	32	32	36
Gansu	10	38	101	33	33	25
Qinghai	5	12	10	39	41	17
Ningxia	6	4	37	34	37	32
Xinjiang	12	19	46	28	22	17
*Northeastern provinces*						
Liaoning	122	175	596	19	20	28
Jilin	36	55	98	27	38	39
Heilongjiang	69	33	76	23	18	21

^a^Total value added of other provinces contributed by the exports of each province

^b^Proportion of the sum of the value added of other provinces contributed by each province’s exports to the sum of the value added contributed by the province’s exports

Based on the percentage of the total value added of other provinces contributed by a province’s exports to the total value added contributed by the exports (i.e., export spillover effect), the export spillover effect of the eastern provinces was greater. Comparing the value added of spillover effects, however, showed that the spillover effect of the eastern provinces was insignificant. The primary reason for this effect is that although the value added of other provinces contributed by the export spillover effects of the eastern provinces was greater, China’s economy, especially the manufacturing industry, is concentrated in eastern provinces with strong agglomeration effects and supporting capacity. Therefore, the production of the products exported from eastern provinces is mainly completed in the local region, which contributes greatly to the eastern provinces’ economies but has a smaller spillover effect on other provinces.

### Analysis of the export spillover effect between provinces

Although the export spillover effect was analyzed in the previous section, the spillover effect between provinces was not clear to date. For example, it was unclear which province was most significantly affected by Guangdong Province’s exports and which other provinces were affected by a specific province’s export spillover effects. Therefore, further analysis was needed. Table 7 shows the three provinces that had the greatest export spillover effects on other provinces in 2007 and related data. Guangdong, Zhejiang, Jiangsu, and Shanghai in the eastern region are the main sources of export spillover effects on provinces in other regions (western, central, and northeastern) in China. Guangdong is the source of the greatest export spillover effects on nearly all other provinces. For example, the three provinces with the greatest spillover export effects on Shaanxi in 2007 were Guangdong, Zhejiang, and Jiangsu. The value added of Shaanxi contributed by these three provinces accounted for 29, 12, and 10 %, respectively, of the sum of the value added contributed by other provinces. The total value added of Shaanxi contributed by the three provinces reached 18.7 billion yuan.

**Table 7:** Three provinces with the greatest export spillover effects in 2007

Region	Three provinces with greatest export spillover effects in 2007^a^	Contribution of the three provinces^b^ with the largest spillover effects (%)			Value added contributed by spillover effect (100 million yuan)^c^
*Eastern provinces*					
Beijing	Shanghai, Guangdong, Tianjin	29	18	10	333
Tianjin	Guangdong, Beijing, Jiangsu	28	12	12	327
Hebei	Guangdong, Zhejiang, Jiangsu	25	19	14	1186
Shanghai	Guangdong, Jiangsu, Zhejiang	26	19	14	345
Jiangsu	Guangdong, Zhejiang, Shanghai	27	18	14	549
Zhejiang	Guangdong, Shanghai, Jiangsu	37	13	12	551
Fujian	Guangdong, Jiangsu, Shanghai	30	11	11	274
Shandong	Guangdong, Zhejiang, Jiangsu	23	22	12	492
Guangdong	Zhejiang, Shanghai, Jiangsu	24	17	16	732
Hainan	Guangdong, Zhejiang, Jiangsu	47	17	12	1300
*Central provinces*					
Shanxi	Guangdong, Jiangsu, Zhejiang	40	18	13	802
Anhui	Guangdong, Jiangsu, Zhejiang	27	16	13	456
Jiangxi	Zhejiang, Shanghai, Jiangsu	39	12	11	258
Henan	Guangdong, Zhejiang, Jiangsu	30	12	11	134
Hubei	Guangdong, Zhejiang, Jiangsu	28	17	16	972
Hunan	Guangdong, Jiangsu, Zhejiang	28	19	17	831
*Western provinces*					
Inner Mongolia	Guangdong, Zhejiang, Jiangsu	27	13	11	221
Guangxi	Guangdong, Jiangsu, Zhejiang	34	11	10	312
Chongqing	Guangdong, Shanghai, Zhejiang	24	17	16	732
Sichuan	Guangdong, Jiangsu, Zhejiang	32	13	12	277
Guizhou	Guangdong, Zhejiang, Jiangsu	27	12	11	32
Yunnan	Guangdong, Zhejiang, Jiangsu	38	15	8	136
Shaanxi	Guangdong, Zhejiang, Jiangsu	29	12	10	187
Gansu	Guangdong, Jiangsu, Zhejiang	29	14	12	148
Qinghai	Guangdong, Shanghai, Jiangsu	30	21	15	285
Ningxia	Guangdong, Shanghai, Zhejiang	24	14	13	394
Xinjiang	Guangdong, Zhejiang, Jiangsu	30	17	16	140
*Northeastern provinces*					
Liaoning	Guangdong, Zhejiang, Jiangsu	22	11	10	9
Jilin	Guangdong, Shanghai, Jiangsu	22	13	10	31
Heilongjiang	Guangdong, Zhejiang, Jiangsu	30	20	12	271

^a^Three source provinces with the largest spillover effects

^b^The percentages of the value added contributed by the exports of the three largest provinces to the value added of the corresponding provinces contributed indirectly by exports

^c^Sums of the value addeds of the corresponding provinces contributed by the exports of the three provinces

Guangdong, Zhejiang, Jiangsu, and Shanghai became the sources of export spillover effects on China’s provinces because the amount of exports of these provinces makes up a large proportion of China’s total exports. These provinces are also located in the heartland of the processing industries in China. Therefore, their exports make a significant contribution to other provinces’ economies.

Guangdong’s exports make the greatest contribution to the value added value added of resource-intensive industries such as mining (A02) and metal smelting and rolling and metal products (A06) in the western region (Table 8). The value added of Inner Mongolia contributed by Guangdong’s exports in 2007 was 25 billion yuan, of which the value added of the mining industry was 8.67 billion yuan and the value added of metal smelting and rolling and metal products was 3.99 billion yuan. This further validates the pattern of regional economic development in China identified earlier: the eastern provinces are the primary exporting areas, and the western provinces are the main suppliers of raw materials for the eastern provinces.

**Table 8:** Value added of different sectors of the western provinces contributed by Guangdong’s exports in 2007 (in 100 million yuan)

Department	Inner Mongolia	Guangxi	Chongqing	Sichuan	Guizhou	Yunnan	Shaanxi	Gansu	Qinghai	Ningxia	Xinjiang
A01	20.4	36.0	5.8	21.2	4.9	9.2	17.5	1.8	0.3	0.5	14.0
A02	86.7	10.4	2.5	7.8	17.4	6.9	59.4	12.6	2.1	2.6	82.5
A03	22.3	21.8	4.0	11.3	6.1	25.4	6.9	0.5	0.1	0.5	2.3
A04	6.7	10.3	3.6	7.5	3.4	4.6	24.1	9.9	0.7	1.6	7.0
A05	2.3	1.3	0.5	0.7	0.5	0.2	0.9	0.4	0.0	0.1	0.2
A06	39.9	27.2	7.8	14.1	11.0	37.9	12.0	24.7	0.1	1.6	3.2
A07	1.0	7.8	38.3	16.2	3.0	2.6	16.3	1.7	0.3	0.9	0.5
A08	23.4	10.6	3.7	5.4	14.8	9.6	7.4	7.2	0.4	3.2	3.2
A09	0.2	0.1	0.2	0.1	0.1	0.1	0.6	0.1	0.0	0.0	0.1
A10	19.5	6.8	3.9	5.2	5.6	6.3	8.7	1.2	0.3	1.0	5.0
A11	16.4	19.3	10.9	10.4	5.8	16.9	19.1	3.9	0.3	2.0	8.7
A12	7.5	3.5	2.2	4.3	4.4	7.2	4.7	1.6	0.2	0.5	2.9
A13	3.8	1.2	1.6	1.6	1.8	2.1	7.4	1.3	0.1	0.3	1.8
Total	250	156	85	106	79	129	185	67	5	15	131

## Conclusions

This paper developed a measurement model for the contribution of exports to the regional economies of a country based on the Chenery–Moses model. The contribution of national and provincial exports to provincial economies in China was measured using China’s MRIO tables for 1997, 2002, and 2007. The following conclusions and policy implications were obtained through the analysis of the measurement results.

First, national exports make significantly different contributions to provincial GDP in different regions in China. The contribution of national exports to the GDP of the eastern provinces was significantly greater than the contribution to the GDP of the provinces in other regions. The contribution of national exports to the GDP of the central and western provinces was small, but the contribution of national exports to the economies of the central and western provinces was significantly greater than the foreign export dependence. Therefore, in the current international market downturn, the eastern provinces, which occupy a higher proportion of foreign trade, must accelerate their transformation to address the negative impacts of the export slump. The central and western provinces must also make full use of their comparative advantages by undertaking an industrial transformation and improving the development environment to address the indirect impacts of the export slump.

Second, each province has a different source of contribution made by exports. The contribution made by exports to the economies of the eastern provinces stemmed mainly from the exports themselves, whereas the contributions made by export to the economies of the central and western provinces (especially the western provinces) stemmed from the export spillover effects of the eastern provinces. This indicates that the eastern provinces are more profoundly integrated into the global industrial chain through the processing trade, while most of the western provinces are not yet integrated into the global industrial chain, but have instead become the suppliers of raw materials for the eastern provinces. Therefore, the western provinces must enhance their endogenous aptitude for economic growth; improve their scientific and technical innovation capability, industrial supporting capacity, and institutional innovation; and change the intensive growth model that relies on resource outputs.

Third, Guangdong, Zhejiang, Jiangsu and Shanghai in the eastern region were the main source of export spillover effects for other provinces in China. In particular, Guangdong was the largest source of export spillover effects and made a great contribution to the value added of resource-intensive industries such as mining in the western region. Therefore, if the exports of Guangdong and the other eastern provinces decrease, other provinces will be affected. This indicates that changes in the international market and foreign trade policy will have an important impact on China’s regional economy.

## Footnotes

1 The causes of China's economic decline also include the internal factor of the transition phase.

2 Many studies on the measurement of foreign trade dependence mainly focus on the choice of the denominator indicator in the dependence calculation equation (GDP or final output) and the calculation standard (price or PPP).

3 For example, in terms of the source, two-thirds of the coal consumed by Beijing is assumed to come from Shanxi and one-third is assumed to come from Hebei. In the IRIO model, the proportion of coal consumed from any given source by different sectors of Beijing may be different, but in the MRIO model, the proportion of coal consumed from any given source by different sectors of Beijing is the same as the assumption.

4 For a detailed description of the MRIO model, please refer to Chenery (1953) and Moses (1955).

5 Import demand has been deducted.
